# Reduced Tumorigenicity of Mouse ES Cells and the Augmented Anti-Tumor Therapeutic Effects under *Parg* Deficiency

**DOI:** 10.3390/cancers12041056

**Published:** 2020-04-24

**Authors:** Yuki Sonoda, Yuka Sasaki, Akemi Gunji, Hidenori Shirai, Tomonori Araki, Shoji Imamichi, Takae Onodera, Anna-Margareta Rydén, Masatoshi Watanabe, Jun Itami, Takuya Honda, Kazuto Ashizawa, Kazuhiko Nakao, Mitsuko Masutani

**Affiliations:** 1 Department of Molecular and Genomic Biomedicine, Nagasaki University Graduate School of Biomedical Sciences, Nagasaki 852-8523, Japan; 2 Department of Gastroenterology and Hepatology, Nagasaki University Graduate School of Biomedical Sciences, Nagasaki 852-8501, Japan; 3 Lab of Collaborative Research, Division of Cellular Signaling, National Cancer Center Research Institute, Tokyo 104-0045, Japan; 4 Biochemistry Division, National Cancer Center Research Institute, Tokyo 104-0045, Japan; 5 Department of Oncologic Pathology, Mie University Graduate School of Medicine, Mie 514-8507, Japan; 6 Department of Radiation Oncology, National Cancer Center Hospital, Tokyo 104-0045, Japan; 7 Department of Clinical Oncology, Nagasaki University Graduate School of Biomedical Sciences, Nagasaki 852-8523, Japan; 8 Center for Bioinformatics and Molecular Medicine, Nagasaki University Graduate School of Biomedical Sciences, Nagasaki 852-8523, Japan

**Keywords:** cancer, poly (ADP-ribose) glycohydrolase, radiosensitization, poly (ADP-ribose) polymerase, ES cells

## Abstract

PolyADP-ribosylation is a post-translational modification of proteins, and poly(ADP-ribose) (PAR) polymerase (PARP) family proteins synthesize PAR using NAD as a substrate. Poly(ADP-ribose) glycohydrolase (PARG) functions as the main enzyme for the degradation of PAR. In this study, we investigated the effects of *Parg* deficiency on tumorigenesis and therapeutic efficacy of DNA damaging agents, using mouse ES cell-derived tumor models. To examine the effects of *Parg* deficiency on tumorigenesis, *Parg^+/+^* and *Parg^−/−^* ES cells were subcutaneously injected into nude mice. The results showed that *Parg* deficiency delays early onset of tumorigenesis from ES cells. All the tumors were phenotypically similar to teratocarcinoma and microscopic findings indicated that differentiation spectrum was similar between the *Parg* genotypes. The augmented anti-tumor therapeutic effects of X-irradiation were observed under *Parg* deficiency. These results suggest that *Parg* deficiency suppresses early stages of tumorigenesis and that *Parg* inhibition, in combination with DNA damaging agents, may efficiently control tumor growth in particular types of germ cell tumors.

## 1. Introduction

PolyADP-ribosylation is the post-translational modification of proteins, and poly(ADP-ribose) (PAR) polymerase (PARP) family proteins synthesize PAR using NAD as a substrate. Poly(ADP-ribose) glycohydrolase (PARG) is involved in the degradation of PAR as a main enzyme. PARP-1 is activated upon DNA damage and is crucial for preserving genome stability [1]. Activated PARP-1 recognizes DNA strand breaks and is involved in base-excision repair, DNA strand break repair pathways [1]. 

The process of polyADP-ribosylation of proteins is tightly regulated by the second enzyme in the metabolic pair: PARG and ADP-ribosyl hydrolase3 (ARH3) [2]. PARG reverses the action of PARP by hydrolyzing the glycosidic bonds of PAR to produce ADP-ribose [3,4]. The catalytic capacity of PARG keeps the polyADP-ribosylation of proteins transient; the conversion rate of PAR is measured in minutes [1]. 

In DNA repair process, PARG is involved in the regulation of base excision repair and DNA strand break repair through the control of XRCC1 function [5]. PARG functional inhibition leads to increase in radiosensitivity in particular types of cells [6,7]. Perturbation of the PARP-1/PARG balance by the over-expression of PARG has been also shown to alter the genome methylation pattern to that of cancer cell types [8].

The importance of PARG in normal development is underscored by the fact that disruption of PARG leads to embryonic lethality [9,10]. PARG enzyme structures have been revealed [11,12,13]. There are several isoforms of PARG, which are produced from a single gene by alterative splicing. The longest (110 kDa, PARG_110_) and most abundant isoform is located in the nucleus, while the 60 kDa isoform is located in the cytoplasm and mitochondria [14,15,16,17]. Disruption of exon 4, which is common to all PARG isoforms, leads to cell lethality [9]. However, disruption of exons 2–3 abrogates expression of nuclear PARG, but still allows expression of the remaining isoforms [18]. Furthermore, deficiency of the full-length isoform of PARG leads to enhanced cytotoxic sensitivity by causing PAR accumulation, induced by menadione, alkylating agents, endo-toxic shock and γ-irradiation [18,19]. 

Upon extensive DNA damage, PARP-1 becomes excessively activated and depletes cellular NAD^+^ to polyADP-ribosylate proteins. PAR accumulation and the depletion of NAD^+^ severely perturb the energy balance of cells and lead to apoptosis. PAR accumulation also triggers AIF (apoptosis inducing factor) activation in mitochondria and induces parthanotos, which can be observed in neuronal and cancer cells [20,21]. In heterozygously knockout mice of *Parg*, suppression of lung cancer development has been reported [22]. 

As described above, the cytoprotective role of PARG through its involvement in DNA repair in normal cells has been suggested, whereas the functional inhibition of PARG is reported to cause sensitization to DNA-damaging agents in cancer cells. Therefore, PARG may be considered both a tumor suppressor and a therapeutic target of cancer. The accumulated evidence thus led us to hypothesize that PARG may impact both cancer development and cancer therapy. Meanwhile, embryonic stem (ES) cells of mice can be useful as experimental systems for tumorigenesis and teratocarcinoma. In this study, using hypomorphic *Parg* knockout ES cells, we investigated the effects of *Parg* deficiency on tumorigenesis and the therapeutic efficacy of DNA damaging agents using ES cell derived tumor models.

## 2. Results

### 2.1. Parg^−/−^ ES Cells Show Delayed Tumor Development

**Figure 1:**
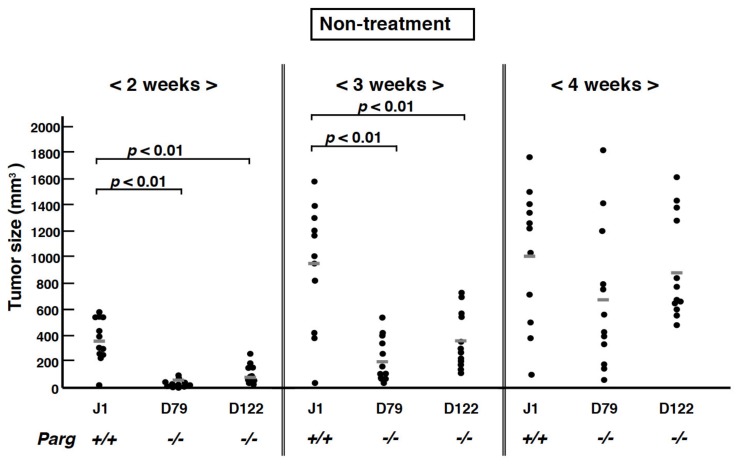
Effect of *Parg* deficiency on tumorigenesis from embryonic stem (ES) cells. In total, 1 × 10^7^ ES cells were inoculated *s.c.* into nude mice and size of tumors was measured weekly. Wild-type, J1. *Parg^−/−^*, D79 and D122.

We previously generated two hypomorphic *Parg^−/−^* ES cell clones, D79 and D122, which retained about 10% residual PARG activity compared to parental wild-type J1 ES cells [7]. The growth rates of these *Parg^−/−^* and J1 ES cells are similar in the absence of DNA damaging agents. To examine the effects of *Parg* deficiency on tumorigenesis, *Parg^+/+^* J1 and two D79 and D122 *Parg^−/−^* ES cells were subcutaneously injected into the flanks of nude mice. Following injection, tumor size development was observed weekly over four weeks. An initial delay of tumor growth was observed at weeks two and three in tumors derived from *Parg^−/−^* ES cells (*p* < 0.01, Figure 1). This effect was observed during only the early phase, as tumor size did not differ significantly between the genotypes at week four. These results indicate that PARG deficiency delays the early onset of tumorigenesis derived from ES cells.

### 2.2. Characterization of Tumor Tissues

**Figure 2:**
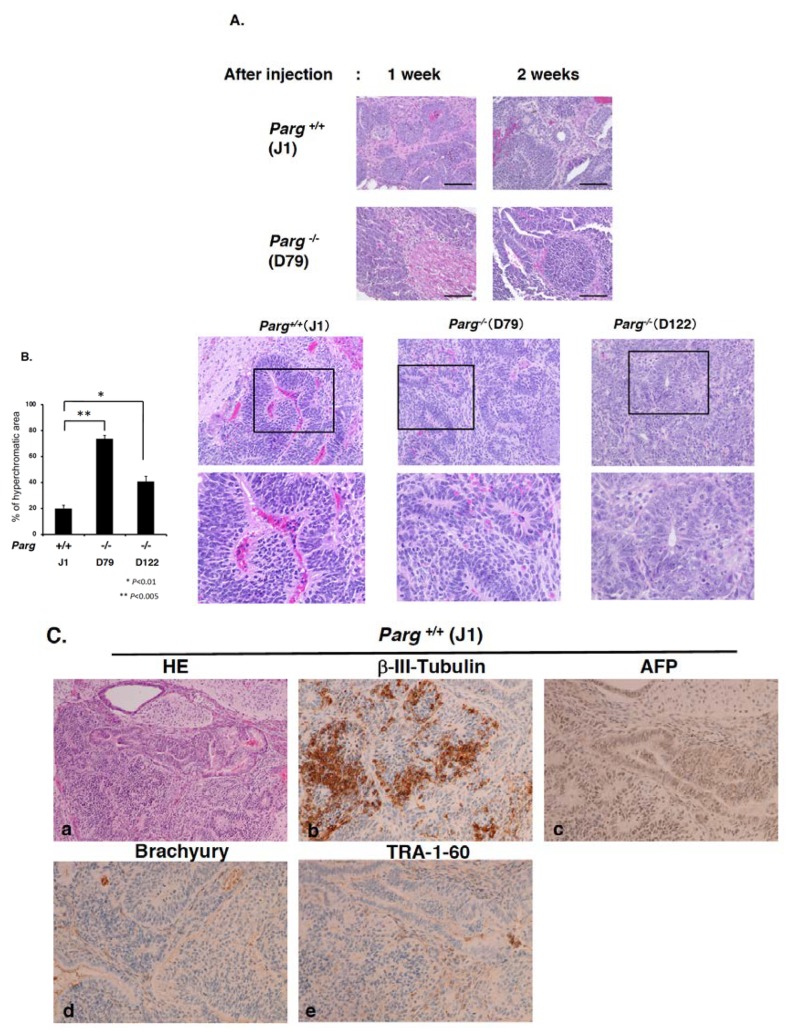

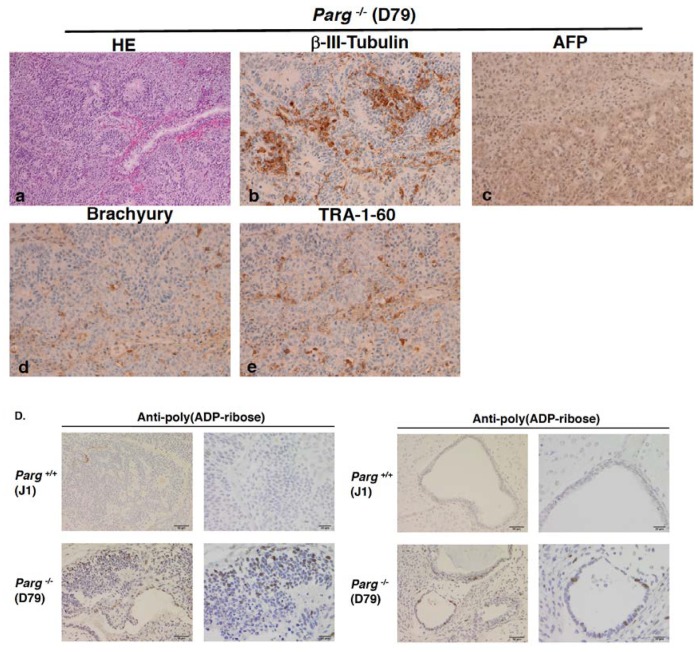
Hematoxylin-eosin staining of tumor tissues derived from mouse ES cells. (**A**) Hematoxylin-eosin staining of tumor tissues derived from mouse ES cells 1 and 2 weeks after injection. Upper panels, wild-type J1. Lower panels, *Parg^−/−^*. (**B**) The left graph shows the percentage of hyperchromatic area of the tumors 4 weeks after injection. Percentage of hematoxylin-positive hyperchromatic area in the total area of tumor section was measured for each tumor. * *p* < 0.01, ** *p* < 0.005. Right panels show the typical hyperchromatic areas of hematoxylin-eosin staining of tumors 4 weeks after injection. Upper panels, ×20 magnification (Squares show magnified regions in the lower panels. Lower panels, ×40 magnification. The tumors showed heterogeneous cell components containing primitive neuroepithelial components and embryonal carcinoma components. (**C**) HE staining and immunostaining of the tumors at 4 weeks with antibodies against b-III-tubulin, ectoderm marker; AFP, endoderm marker (×20 magnification). Hematoxylin-eosin staining, ×10 magnification. The mixed staining pattern of ectodermal and endodermal markers was observed in hyperchromatic regions of *Parg^−/−^* tumors at 4 weeks. (**D**) Immunostaining of the tumors at 4 weeks after injection with antibody against anti-PAR. Right panels in D are magnified images, Bars, 50 mm (left panels in D), 20 mm (right panels in D). PAR staining was observed occasionally in the cell nuclei in the *Parg^−/−^* tumor but not in the *Parg^+/+^* tumor.

To further characterize the tumorigenesis, histological evaluation of HE-stained tumor sections was carried out. Comparison of tissues and cell type are summarized in Table 1. Tumors observed four weeks after injection into nude mice showed ectodermal, mesodermal and endodermal tissue derivatives. Both undifferentiated and differentiated germinal components were detected. Histopathological examination at four weeks identified all tumors as immature teratoma, partially accompanying embryonal carcinoma components. Microscopically, the tumors showed heterogeneous components, especially containing immature neuroectodermal tissue in the form of primitive neuroepithelial rosettes and tubules (Figure 2B,C). 

We previously observed that tumors derived from *Parp1^−/−^* ES cells showed differentiation into trophoblast lineages, including trophoblast giant cells [23]. Microscopic findings from the tumors derived from *Parg^−/−^* ES cells showed no such components, suggesting that in the hypomorphic *Parg* deficient state, marked differentiation alterations did not occur (Table 1).

### 2.3. Time Course Analysis of Tumorigenesis

To evaluate the defect in early stage tumorigenesis under *Parg* deficiency, further histological analyses were performed on sections of tumor tissues (Figure 2A,B). At one and two weeks after injection, tumors derived from *Parg^−/−^* ES cells showed a higher tendency of necrosis. The density of tumor cells and stromal cells appeared to be lower in the *Parg^−/−^* tumors. As shown in Figure 2B, comparison of percentage of hematoxylin-positive regions in the tumors at four weeks (Figure S1) showed the augmented hematoxylin-positivity, namely hyperchromatic areas (typical areas are shown as Figure 2B), in *Parg^−/−^* tumors with a statistical significance. It may suggest that the chromatin density of the cells was higher, possibly reflecting differences in the chromatin state or cell properties. 

To characterize the properties of differentiated cells and hyperchromatic components further, we performed the immunostaining analysis for the tumors at four weeks with antibodies against beta-III-tubulin, ectoderm marker; AFP, endoderm marker; TRA-1-60, pluripotent marker, and Brachyury, mesoderm marker. As shown in Figure 2C, immunohistochemical analysis showed beta-III tubulin-positive staining of immature neuroepithelial tissues in both wild-type and *Parg^−/−^* tumors. It is, therefore, implied that hyperchromic regions may consist of both ectodermal and endodermal differentiated tissues. The pluripotent marker TRA-1-60 showed higher tendencies of diffused staining in the stromal components of the *Parg^−/−^* tumor compared with the wild-type tumor. For the mesoderm marker Brachyury, a higher tendency of staining was also observed in the cell components of *Parg^−/−^* tumor. On the other hand, AFP-positive staining patterns of teratocarcinoma and immature glandular components were similar between *Parg^−/−^* and wild-type tumors.

We also analyzed whether PAR accumulation occurs in *Parg^−/−^* tumors, due to hypomorphic Parg activity. As presented in Figure 2D panels, PAR staining was observed occasionally in the cell nuclei of the *Parg^−/−^* tumors but not in the *Parg^+/+^* tumor. This elevated level of PAR confirmed the defect of Parg activity.

### 2.4. Augmented Anti-Tumor Therapeutic Effects under Parg Deficiency

**Figure 3:**
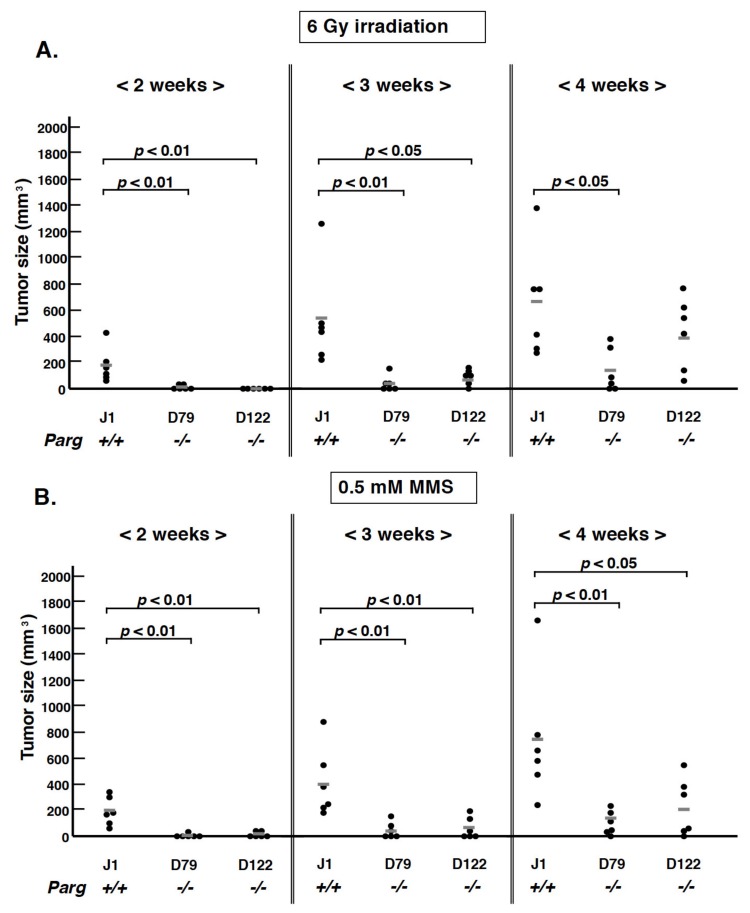
Effect of *Parg* deficiency on tumorigenesis from ES cells pretreated with MMS or γ–irradiation before injection into nude mice. ES cells treated with γ–irradiation at 6 Gy (**A**) or 0.5 mM MMS (**B**) and cultured for 3 h, respectively, were inoculated *s.c.* into nude mice. Tumor growth was observed for four weeks. The bars show the mean size.

Previous reports describe that *Parg _110_* deficient animals are more sensitive to MMS treatment and γ-irradiation compared to wild-type mice [18,19]. Hypomorphic *Parg^−/−^* ES cells and particular human cancer cells with *PARG* knocked down also showed increased sensitivity to DNA-damaging agents, such as alkylating agents and γ-irradiation. Therefore, we first treated ES cells in vitro with MMS and γ-irradiation, and three hours later, the cells were subcutaneously injected to nude mice to observe the tumor growth. As shown in Figure 3A,B, reflecting the in vitro higher sensitivity of *Parg^−/−^* ES cells, the tumor growth is delayed for at least 2–3 weeks after treatment with both MMS and 6 Gy γ-irradiation. 

**Figure 4:**
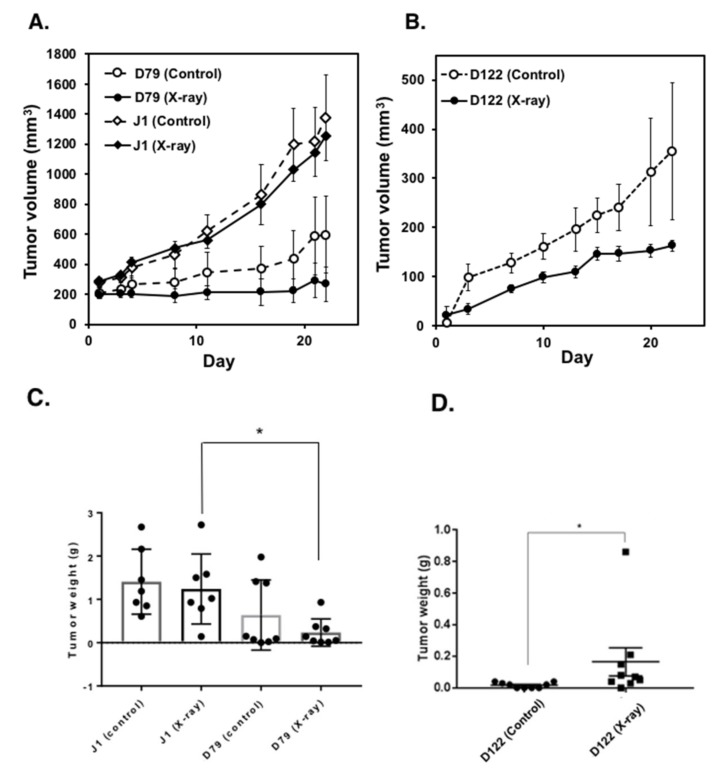
Effect of *Parg* deficiency on therapeutic efficacy of X–irradiation on tumors derived from ES cells. Tumors of one of the hinder leg pair were subjected to local X–irradiation with a single dose at 7 Gy ((**A**,**C**): *n* = 7 for wild-type (J1), *n* = 8 for *Parg^−/−^* (D79). (**B**,**D**): *n* = 9 for wild-type (J1) and *Parg^−/−^* (D122)). Tumor growth of non-irradiation controls (Control) was monitored for non-irradiated side of hinder leg pair. Tumor growth was observed for 22 days. (**A**,**B**) Tumor growth curve, Mean ± S. E. (**C**,**D**) Measured tumor weight 22 days after injection of ES cells, Mean ± S. E. (**C**) Statistical analysis was carried out with Turkey’s test. * *p* < 0.05. (**D**) Statistical analysis was carried out with Mann–Whitney *U*-test. * *p* < 0.01.

Next, we carried out therapeutic models of local X-irradiation using tumors derived from wild-type and *Parg^−/−^* ES cells. As shown in Figure 4A–D, when the tumors derived from *Parg^−/−^* ES cells D79 and D122 were X-irradiated at the single dose of 7 Gy, they showed a delay in tumor growth. In contrast, when the tumors derived from wild-type J1 ES cells were X-irradiated, delay in tumor growth was not clearly observed. It is thus suggested that the therapeutic efficacy of X-irradiation could be higher in the tumors harboring *Parg* deficiency.

These results suggest that Parg inhibition in combination with DNA damaging agents may efficiently control tumor growth in particular types of germ cell tumors.

## 3. Discussion

Our findings demonstrate that *Parg* deficiency delays the early onset of cancer in vivo in teratocarcinoma model. This suggests that PARG might be an attractive therapeutic target in cancer control of germ cell tumors. The therapeutic model used in this study showed that X-irradiation is more effective in tumors with *Parg* deficiency compared to wild-type cells.

ES cells are derived from normal cells and their tumor models are phenotypically close to teratocarcinoma and teratoma. It is reported that tumorigenicity of ES cells is driven by oncogene action of *E-Ras* gene [24], and the genes including PI(3)K [24] and *c-Jun* [25], *Cox1/2* [26] and *Oct3/4* [27] are involved in the tumorigenesis process. The present study implies that *Parg* could be a candidate target for the suppression of tumorigenesis at early stages.

In the subcutaneous injection model of ES cells used herein, ES cells should be grown in spheroids or attached to extracellular matrix in subcutaneous regions. During the stressed period of early tumorigenesis, the presence of *Parg* was suggested to enhance cell survival. In cultured *Parg^−/−^* ES cells, the PAR degradation activity was decreased to 10% of wild-type ES cells [7]. We observed that PAR staining was observed occasionally in the cell nuclei of the *Parg^−/−^* tumors but not in the *Parg^+/+^* tumors. This accumulation of PAR confirmed the defect in *Parg* activity. The infrequent accumulation of PAR may explain the advance of tumor growth in *Parg^−/−^* tumors at four weeks, as efficient PAR degradation may be necessary for proliferation. The pluripotency marker staining of *Parg^−/−^* tumors at four weeks was also observed in a scattered manner, suggesting that the presence of a majority of differentiated tissues could be related to the infrequent accumulation of PAR in *Parg^−/−^* tumors, as differentiated cells are reported to have low levels of PAR compared with proliferating cells [1]. The occasional increase in PAR staining in *Parg^−^/^−^* tumors could be possibly due to the presence of S-phase cells with stalled replication forks [28], leading to a lower proliferation activity. Another possibility could be an increased expression of other PAR degradation enzymes, such as ARH3 in the differentiated tumors. The clarification of the relationship between PAR accumulation and cell proliferation needs to be addressed in further studies. 

When we added further stress with alkylating agents and γ-irradiation to ES cells (Figure 3A,B), tumor development was further suppressed under *Parg* deficiency. This was consistent with our previous observation that when *Parg* was inhibited in ES cells, caspase-dependent apoptosis was enhanced with S-phase arrest and PAR accumulation after MMS treatment [29]. 

**Figure 5:**
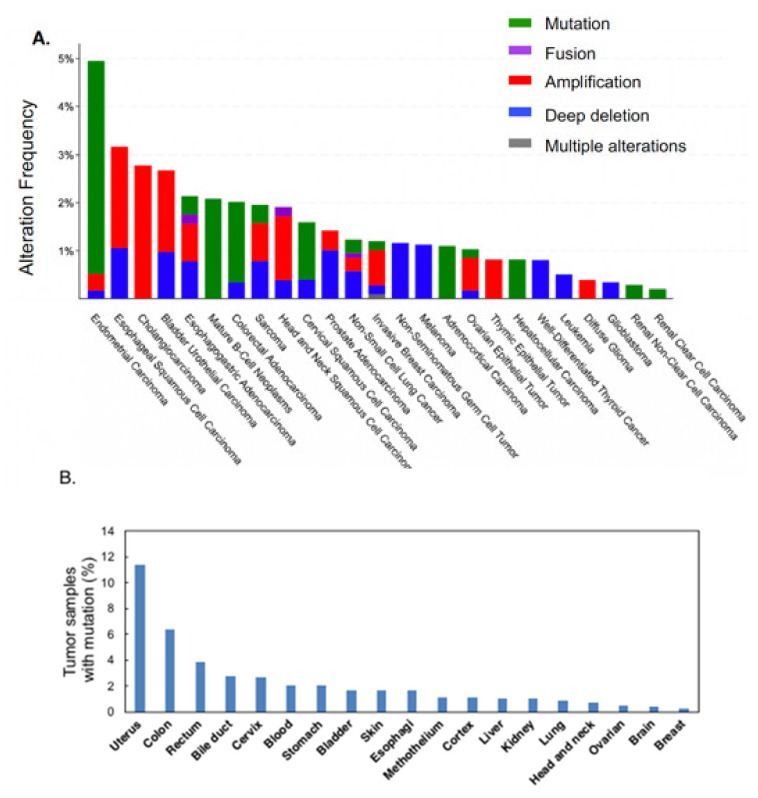
*PARG* mutation frequencies in human cancers. (**A**) The percentage of nonsynonymous mutation of *PARG* in each cancer in the TCGA database [40]. (**B**) The percentage of nonsynonymous substitution mutation of *PARG* in each cancer (total 165 mutations were listed in tumor samples from 8018 patients.) in the CanSAR database [41].

In diseases, PARP-1 has been shown to be involved in stroke, ischemia diabetes and other inflammatory diseases [30]. PARP-1 is frequently overexpressed in various types of cancers [31,32,33]. As PARG has an opposing enzymatic activity to PARP-1, it is reasonable to hypothesize a similar involvement in disease categories. The involvement of PARG in cancer development has not been extensively studied. However, recent genome sequencing approach accumulated the information of *PARG* gene mutations in various types of cancer. As shown in Figure 5, the data from TCGA database (A) and CanSAR database (B) showed that *PARG* gene mutations are present in particular types of cancers and of note, deep deletions could be observed in non-seminomatous germ cell tumors, melanoma, and well-differentiated thyroid cancers, possibly indicating PARG-deficient state. On the other hand, esophageal squamous cell carcinoma, cholangiocarcinoma, bladder urothelial carcinoma, thymic epithelial tumors and diffuse glioma show amplification of *PARG* gene, possibly suggesting the overexpression of PARG. In fact, PARG activity is reported to be high in C6 glioma tumor cells [34]. With further clinical and basic studies, PARG-deficiency and PARG-overexpression may become useful biomarkers for therapeutic selection and monitoring. 

Functional inhibition of PARG in pancreatic cancer cells, which has p53 pathway inactivation, enhanced the necrotic cell death after MMS treatment [29], suggesting that PARG could be a therapeutic target in certain types of cancer cells. It is reported that ovarian cancer cells show differential sensitivity to PARG and PARP inhibitors, and cells with replication vulnerability show persistent replication fork stalling and replication catastrophe, with treatment of the PARG inhibitor and sensitization to CHK1 inhibitor [35]. 

Whilst the hypomorphic ES cells used in this study show PARG activity of residual 10%, the cell growth was not reduced compared to wild-type ES cells [29], suggesting that ES cells may not be replication vulnerable cells. *Parg* deficient ES cells also showed a higher sensitivity to cisplatin, but not to topoisomerase I inhibitor, campthothecan and hydrogen peroxide [36].

PARG is involved not only in the DNA repair pathway but also in various cell physiological processes, including epigenetic regulation [8], microRNA regulation and RNA splicing. Pancreatic ductal adenocarcinoma cells expressing pro-oncogenic mRNA stability factor HuR (ELAVL1) show sensitization to oxaliplatin and 5-fluorouracil through persistent PARylation [37]. Silencing of PARG by shRNA decreased the proliferation rate twofold over wild type LoVo colon cancer cells [38]. PARG function was also suggested to be involved in MAP kinase cascade regulation, as implicated by the synthetic lethal effect under *PARG* and *DUSP22* double dysfunction [39]. The mechanism for delayed tumor formation process under *Parg* deficiency in ES cells should be further analyzed from multiple physiological aspects. 

## 4. Materials and Methods

### 4.1. Cell Culture

The cell lines used were cultured at 37 °C in a humidified incubator, where CO_2_ levels were kept at 5%. ES cells were cultured in Dulbecco’s modified Eagle’s medium (Life Technologies Corp., Carlsbad, CA, USA) supplemented with 20% fetal bovine serum, non-essential amino acids (Life Technologies Corp.), 55 µM β-mercaptoethanol, 0.3 mM each of adenosine, guanosine, and thymidine, 0.1 mM uridine, and 1 × 10^3^ U/mL mouse LIF (Chemicon International Inc., Temecula, CA, USA) on gelatin-coated dishes (Asahi Glass Co. Ltd., Tokyo, Japan). Mouse ES cell J1 established from 129Sv/J mouse was provided by Dr. Ochiya of National Cancer Center Research Institute [42]. Hypomorphic two *Parg^−/−^* ES cell clones, D122 and D79, which we previously established [7], were used in this study. MMS (Sigma-Aldrich Co., St. Louis, MO, USA) was prepared in saline before use and sterilized by filtration.

### 4.2. Tumorigenesis Analysis

ES cells (J1, D122 and D79) cultured in the absence of a STO feeder layer [42] were trypsinized and 1 × 10^7^ cells (Figure 1 and Figure 3) or 5 × 10^6^ cells (Figure 4) were subcutaneously injected into both legs of male BALB/c-nu/nu mice (CLEA Japan, Tokyo). The growth of tumors was measured continuously every 3–4 days. Four weeks after injection of ES cells, mice were euthanized and each tumor was histologically analyzed. The tumor volumes were calculated with the following formula: (smallest diameter)^2^ × (largest diameter)/2. For the time course experiment, mice were euthanized at 7 and 14 days after subcutaneously injection of ES cell. Local X-ray irradiation of the tumors on hind legs were carried out with an X-ray machine (CP-160, 160-kVp, Faxitron X-Ray Corp., Tucson, AZ, USA) using radiation shield of lead. Tumors of one of the hind leg pair were subjected to local X–irradiation with a single dose at 7 Gy (Figure 4, A and C: *n* = 7 for wild-type (J1), *n* = 8 for *Parg^−/−^* (D79). B and D: *n* = 9 for wild-type (J1) and *Parg^−/−^* (D122)). Tumor growth of non-irradiation controls (Control) was monitored for non-irradiated side of hind leg pair. All animal experiments were approved by the Institutional Animal Experiment Committee of National Cancer Center Research Institute (T05-053-C01, A377-16, T17-053). All animal works were conducted according to relevant national and international guidelines for animal welfare.

### 4.3. Histological Analysis

After resection of the tumors, they were fixed in neutralized 10% formalin solution and embedded in paraffin blocks using standard procedures. Paraffin sections were stained with hematoxylin-eosin (HE), and histopathological analysis was performed under a light microscopic observation. Tissue sections mounted on slides were also subjected to immunostaining after deparaffinization and rehydration, and antigen retrieval, according to the manufacturer’s protocol. Antibodies used in this study were anti-beta-III-tubulin (Abcam, ab264113, Cambridge, UK), anti-AFP (α-fetoprotein, Pierce, PA5-21004, Shreveport, LA, USA), anti-TRA-1-60 (Santa Cruz, sc-21705, Dallas, TX, USA), Brachyury (Santa Cruz, sc-374321, Dallas, TX, USA) and anti-PAR (BD Pharmingen, Franklin Lakes, NJ, USA). After several washes with PBS, bound antibodies were visualized using 3, 3′-diaminobenzidine according to the manufacturer’s protocol. The sections were counterstained with hematoxylin and mounted.

### 4.4. Statistical Analysis

Statistical analysis was carried out by the One-way ANOVA test, Mann–Whitney *U* tests, Turkey’s test and Student *t*-tests using either SPSS Statistics of Macintosh version (IBM corporation, Armonk, NY, USA), JMP (SAS Institute Inc. Cary, NC, USA) or GraphPad Prizm7 (GraphPad Software Inc., San Diego, CA, USA).

## 5. Conclusions

In conclusion, our results demonstrate that *Parg* deficiency delayed the onset of tumor formation of ES cells and augmented anti-tumor therapeutic effects of DNA-damaging agents, including alkylating agents and x- ray irradiation. These results suggest that the inhibition of PARG is likely to be an option for the therapeutic and prophylactic target of cancer control. Some specific inhibitors for PARG have been reported [43,44,45], although clinical studies for therapeutic agents are yet to take place. PARG inhibitors, in combination with DNA-damaging agents, may efficiently suppress tumor growth in particular types of germ cell tumors.

## Figures and Tables

**Table 1:** Tissue components in tumors from *Parg^+/+^* and *Parg^−/−^* ES cells.

Tissue Type	*Parg^+/+^*(J1)	*Parg^−/−^* (D79)	*Parg^−/−^* (D122)
Embryonal carcinoma	+	+	+
Hemorrhage	−	−	−
Trophoblast giant cells	−	−	−
*Ectodermal derivatives*			
Primitive neuroepithelium	+	+	+
Mature neural tissue	+	+	+
Keratinized epithelium	+	+	+
*Mesodermal derivatives*			
Cartilage	+	+	+
Bone	+	+	+
Blood vessel	+	+	+
Lymphocyte and blood cell	+	+	+
Muscle	+	+	+
*Endodermal derivatives*			
Ciliated epithelium	+	+	+
Gut epithelium	+	+	+

## Supplementary Materials

The following are available online at https://www.mdpi.com/2072-6694/12/4/1056/s1, Figure S1: Hematoxylin-eosin staining of tumor tissues derived from mouse ES cells 4 weeks after injection.
