# Supplementary material for: Reduced Tumorigenicity of Mouse ES Cells and the Augmented Anti-Tumor Therapeutic Effects under Parg Deficiency
Source: Cancers (Basel). 2020 Apr 24;12(4):1056. doi: 10.3390/cancers12041056 (PMC7226256; doi:10.3390/cancers12041056)
Supplement: Supplementary file 1 [file cancers-12-01056-s001.pdf]

cancers

MDPI

Article

Reduced Tumorigenicity of Mouse ES Cells and the Augmented Anti-Tumor Therapeutic Effects under *Parg* Deficiency

Yuki Sonoda, Yuka Sasaki, Akemi Gunji, Hidenori Shirai, Tomonori Araki, Shoji Imamichi, Takae Onodera, Anna-Margareta Rydén, Masatoshi Watanabe, Jun Itami, Takuya Honda, Kazuto Ashizawa, Kazuhiko Nakao, Mitsuko Masutani

Supplementary Materials

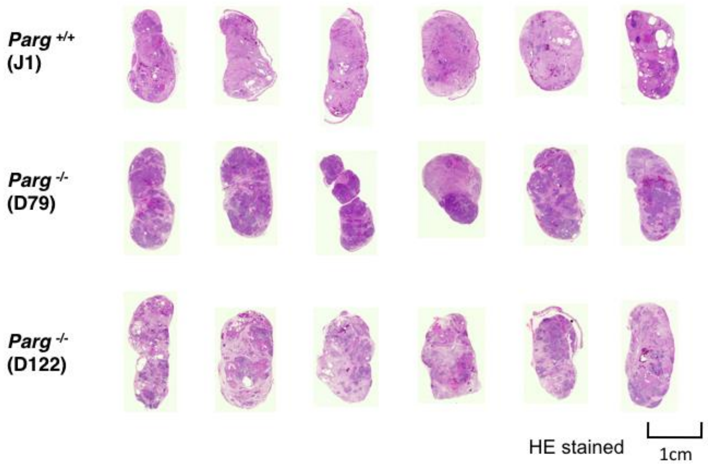

Figure S1. Hematoxylin-eosin staining of tumor tissues derived from mouse ES cells 4 weeks after injection.

© 2020 by the authors. Licensee MDPI, Basel, Switzerland. This article is an open access article distributed under the terms and conditions of the Creative Commons Attribution (CC BY) license (http://creativecommons.org/licenses/by/4.0/).
